# Supplementary material for: Learning curve and surgical outcome of robotic assisted colorectal surgery with ERAS program
Source: Sci Rep. 2022 Nov 29;12:20566. doi: 10.1038/s41598-022-24665-w (PMC9709162; doi:10.1038/s41598-022-24665-w)
Supplement: Supplementary file 2 — Supplementary Information 2. [file 41598_2022_24665_MOESM2_ESM.zip › RAL Raw data-Ñ[▒K/119/OP_ñw╝╨░O▒Kñσ.pdf]

正式摘要(電子簽章：000000227600930)

請選擇...reporter

臺中榮民總醫院

Operation Note

[redacted]

- 手術日期：2021/10/14
- 手術醫師：VS 蔣鋒帆，CR 陳瑋夫，R 陳凱祥，R 林順鴻
- Anesthesia : GE
- Position : Lithotomy
- Aseptic agents : Soap

Diagnosis

- Pre-operative diagnosis : RECTUM Ca
- Post-operative diagnosis : Ditto.s/p robotic APR

Surgical Indication

- for curative resection

Operation

- 1. Robotic (XI) assisted APR
  2. Intraoperative colonoscopy : (-)
  3. High ligation (+) : At IMA root. Left colic artery and IMV were also ligated
  4. Take down from the splenic flexure : (-)
  5. Robotic endoGIA (45-4.1) X1
  6. Proximal cut end: >10 cm
  7. two Fr. 12 penrose was placed from perineum into pelvic cavity
  8. ADHESION BARRIER FILM was placed into pelvic cavity

Finding

- 1. A 2x1cm Borrmann type II tumor at dentate line
  2. No other gross tumor or polyp was found.
  3. There was no palpable liver metastasis or visible peritoneal seeding.

Procedure

- 1. General endotracheal anesthesia was induced and then the patient was placed in modified Trendelenburg lithotomy position.
  2. Preoperative antibiotics were given. A Foley catheter and nasogastric tube were placed.
  3. The abdomen was prepped and draped in standard sterile fashion.
  4. A small vertical incision was made above the umbilicus and a 8 mm trocar was inserted into the peritoneal cavity by open method. Peumoperitoneum was established with CO2 inflation.
  5. Laparoscopy was inserted and then other two 8mm trocars were inserted over LUQ, RLQ of abdomen. Another 12mm trocars was inserted over RLQ area, and the other assist-port 5mm troca was inserted on RUQ. The operative findings were mentioned as above.
  6. The sigmoid colon was held by grasper and the peritoneum overlying the mesentery was scored with electrocautery.
  7. The inferior mesentery artery was identified, ligated above the left colic artery with endoclips, and transected.
  8. Dissection was done above the Gerota's fascia and toward the peritoneal attachments.
  9. The colon was mobilized from retroperitoneum overlying the sacral promontory and into the pelvis. The incision was extended laterally and medially across the peritoneal reflection.
  10. The mesorectum was then mobilized.
  11. Distal descending colon and rectum were mobilized.
  12. The mesorectum was then mobilized using sharp dissection until the levator ani muscle was

- identified.
  13. The proximal D colon was divided with Robotic endoGIA (45-4.1) X1 and pull into pelvic
  14. An elliptical incision was made encompassing the anus and extending up to posterior wall.
  15. Dissection then progressed through through subcutaneous tissues circumferentially.
  16. Hemostasis was achieved with electrocautery.
  17. The abdomen was entered posterior to the anus in the midline.
  18. The levators were then divided with electrocautery beginning posteriorly and progressing anteriorly.
  19. The specimen was delivered through this posterior incision into the perineal field.
  20. The remaining anterior attachments were then severed and the specimen was removed.
  21. Hemostasis was achieved in the pelvis and perineal wound.
  22. Two 12mm penrose was placed at pelvis via perineum.
  23. After placing the ADHESION BARRIER FILM into pelvic cavity. The peritoneum over pelvis was closed with No.1 Vicryl and The skin was closed with 2-0 Nylon.
  24. The fascia of was closed with #1 Vicryl and the skin was closed with interrupted 3-0 Nylon.
  25. The colostomy was then matured with multiple interrupted suture of 4-0 Monocryl.
  26. The patient tolerated the procedure well and was brought to the POR in stable condition
- Estimated blood loss: minimum
- 器械數量: 正確
- 紗布數量: 正確

Image

- PAINT
  - abdomen

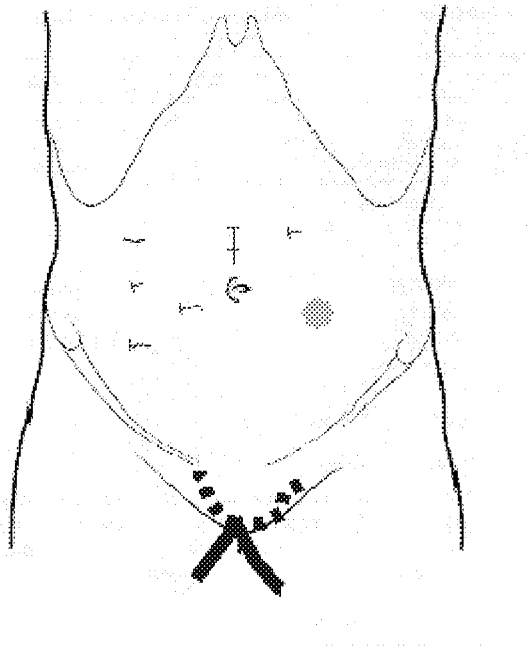

  - colon & rectum

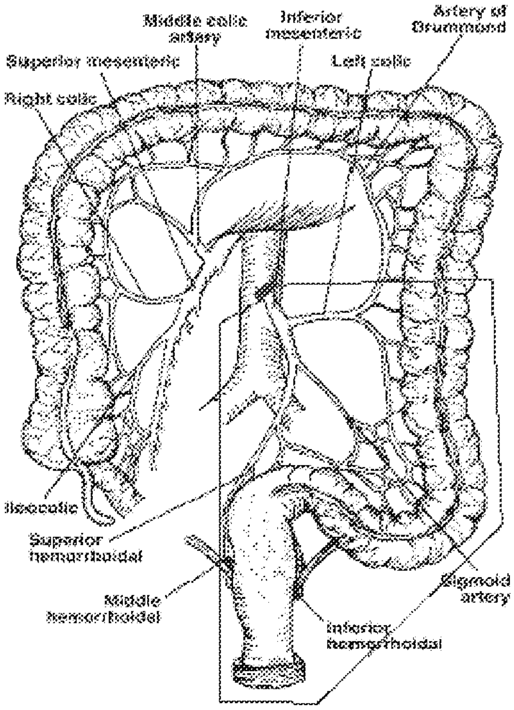

主任 2062F蔣鋒帆 / 醫師 2310K陳瑢夫

正式摘要(**電子簽章：000000227600930)**

請選擇...reporter

doctorRank:B
